# Psychometric evaluation of the characteristics of resilience in sports team inventory in China

**DOI:** 10.1371/journal.pone.0234134

**Published:** 2020-06-12

**Authors:** Yongtao Yang, Yajing Li, Yanlin Sun

**Affiliations:** 1 Institute of Sports Training Science, Tianjin University of Sport, Tianjin, China; 2 School of Physical Education and Educational Science, Tianjin University of Sport, Tianjin, China; University of Florence, ITALY

## Abstract

This study examines the reliability and validity of the Characteristics of Resilience in Sports Teams Inventory (CREST) in Chinese team athletes. A sample of 659 athletes (male = 355, female = 304) aged from 16–34 (*M* = 20.08, *SD* = 2.98) participated in this study. The scale was translated into Chinese using forward and back translation procedures by two independent translators. Questionnaires were administered online. Data was analysed using SPSS 19.0 and Mplus 6.0. Results showed that the items were understood by Chinese team sport athletes. Exploratory factor analysis showed that the Chinese version of CREST had two sub-dimensions as it was in the original scale. Confirmatory factor analysis further demonstrated that the two-structure model was confirmed in the Chinese team sports context. The Cronbach’s alpha values of the scale was 0.842, and the test–retest reliability coefficient of one-month interval was 0.836. It is concluded that the Chinese version of CREST can be used as a valid and reliable tool to assess team resilience in China and can be helpful and applicable in helping sports psychologists understand team resilience. Future studies should further examine the psychometric properties of the scale among world-class athletes and develop a team resilience measurement tool based on Chinese traditional culture.

## Introduction

Pressure is a common situation in many high achievement situations. Athletes in particular, always need to attain and sustain excellent performance under tremendous pressure. Therefore, an important challenge to success for athletes is to positively adapt to the pressure [1, 2] both in training and competition. However, why do some athletes cope with pressure better than others and win, whereas others cannot cope with pressure and subsequently lose? Over the past two decades, sports psychologists have focused on this phenomenon and found many psychological characteristics that help athletes cope with pressure effectively.

One of the psychological characteristics under study is resilience [3], which has positive, preservative and long-lasting effects in coping with pressure, and is an important mechanism for athletes to achieve outstanding performance [4]. Researchers found that highly resilient athletes continue to be more focused, optimistic, exceedingly motivated, and reach for their goals when facing pressure even under failure. They can accept that their effort does not always lead to success and are more receptive to failure. Moreover, highly resilient athletes believe in their ability to influence the course of events, look for the positive meaning of pressure and failure, and believe in their ability to control [5]. Therefore, highly resilient athletes are more likely to succeed under tremendous pressures in competition. Athletes, coaches and news organisations often use psychological resilience to explain athletes’ excellent performance.

The definition of resilience comes from the Latin word “resiliere,” which means bouncing back [6]: it reflects a person's ability to cope with, and adapt to pressure. Resilience is a multidimensional characteristic that varies in different situations, such as time, age, gender, culture, individual life circumstances, and so on [7]. Resilience can be: a buffering factor that protects individuals from psychotic disorders [8], a dynamic process encompassing positive adaptation under tremendous adversity or pressure [9], and a personality characteristic that moderates the negative effects of stress and promotes adaptation [10]. These definitions of resilience show that some researchers investigated resilience as a trait while others viewed resilience as a process. Indeed, some researchers proposed that resilience is not something that individuals innately possess [11], it is a phenomenon that results from the interaction between the individual and the environment [12]. However, there is still no generally agreed definition, which leaves researchers unable to identify a consensus-driven operationalisation of resilience [11].

In addition, many researchers did not distinguish between resilience in the individual and resilience at team level. For example, Lee and Cranford [13] defined resilience as “the capacity of individuals to cope successfully with significant change, adversity, or risk”. Leipold and Greve [14] viewed resilience as “an individual’s stability or quick recovery (or even growth) under significant adverse conditions”. Fletcher and Sarkar [1] defined resilience as “the role of mental processes and behavior in promoting personal assets and protecting an individual from the potential negative effect of stressors”. These concepts mean that researchers have always focused on resilience from an individual perspective, while less attention has been given to whether the individual construct is suitable for measuring and explaining the resilience at team level. Based on the concept of individual level, researchers may omit some important information, leading them to fail to access team resilience or even make incorrect inferences. In fact, researchers found that teams often faced stressors, such as poor interaction quality, poor communication channels, lack of back-up behaviour, and negative organizational culture [6], and group tensions, blame, and sudden slumps in collective performance [15]. These stressors may be different to those that individuals have experienced in their life, especially when a team has experienced a poor performance or failure. A team’s resilience is likely to differ depending on the nature of the situation at different time, such as continuous failure and continuous success. Therefore, a focus on the individual’s resilience is not enough [16] in resilience study. Researchers should also pay attention to team resilience from the social-ecological perspective within the team environment, especially in team sports which have complex interpersonal relationships, communications and competitive pressures. This could help sport psychologists to better understand how teams can sustain optimum performance under pressure.

Team resilience may play an important part in positive team level uptake that aids in the repair and rebound of teams when facing potentially pressured situations [17]. Bennett, Aden [18] proposed that resilience may be viewed “as much a social factor existing in teams as an individual trait”. Carmeli, Friedman [19] defined team resilience as “a team’s belief that can absorb and cope with strain, as well as a team’s capacity to cope, recover and adjust positively to difficulties”. Maynard and Kennedy [20] viewed team resilience as “an emergent state, given the idea that resilience is dynamic”. Morgan, Fletcher [21] also defined team resilience in elite sport as a “dynamic, psychosocial process that protects a group of individuals from the potential negative effects of stressors they collectively encounter”. They further identified four main characteristics of team resilience: group structure, mastery of approaches toward adversity, social capital and collective efficacy. A follow-up study by Morgan, Fletcher [22] revealed developmental antecedents of team resilience and pointed out that coaches could prepare the team for upcoming adversities in four ways, based on the four main characteristics of team resilience. This is the first of two studies focusing on team resilience in sports. Overall, team resilience is an emergent state which suggests underlying dynamic properties [6, 20], and is consistent with the characteristics of sports situations.

Resilience assessment is an important topic for researchers to focus on. Some self-report measurement tools have been developed to evaluate resilience during the past two decades [23–27]. The Connor-Davidson Resilience Scale (CD-RISC) developed by Connor and Davidson [7] is one of the assessment tools which has been widely used in different cultural backgrounds. Campbell-Sills and Stein [28] examined the psychometric properties of the Connor-Davidson Resilience Scale (CD-RISC) and developed the 10-item version of CD-RISC. Some researchers have tested the reliability and validity of the 10-item version of CD-RISC across different cultures [5, 29–32]. However, researchers suggested that the important problem in resilience measurement is not the resilience construct, but the lack of agreement on how resilience should be operationalized [33]. It is a commonly found challenge associated with the operationalisation of the latent psychological structure of resilience [11]. At present, a notable absence in the measurement of psychological resilience is the lack of attention to social-environmental and demographic information. Independent predictors of resilience such as socio-contextual variables are of particular importance, because these variables may exert a cumulative influence on resilience [11], especially in team contexts.

In recent years, the value of resilience to athletes’ performance has received increasing attention. It is not surprising that resilience has become a new and important concept in sport psychology [9, 22, 34]. Researchers especially highlighted the need to measure resilience for athletes and further develop measurement tools based on three pivotal components: adversity, positive adaptation, and protective factors [23]. These elements are important for measurement tool development and could help sport psychologists to better understand resilience. Gucciardi, Jackson [35] explored the dimensionality and measurement invariance of the CD-RISC in Australian cricketers and suggested that the CD-RISC can help researchers identify the antecedents and outcomes of resilience in sport situations. However, other researchers proposed that these assessment tools are not suitable for the evaluation of the resilience level of athletes who actively utilise and optimise a constellation of characteristics when facing pressure and adversity in order to improve sport performance [1, 23].

With regard to the value of resilience on sport performance, there are few self-report tools focused on the measurement of team resilience in a sport context. Based on the definition of team resilience proposed by Morgan, Fletcher [21], Decroos, Lines [2] developed the Characteristics of Resilience in Sports Teams Inventory (CREST) to assess team resilience in sports. CREST has two important structures, which reflect the team’s ability to demonstrate resilient characteristics (DRC) and vulnerabilities under pressure (VNP). CREST assesses team resilience from four dimensions: group structure, mastery approach, social capital, and collective efficacy. It is a 20-item tool, scored on a seven-point Likert scale, ranging from strongly disagree to strongly agree, including twelve positively worded items, reflecting the characteristics of resilience and eight negatively worded items, reflecting vulnerabilities under pressure. Results suggested that the CREST scale assesses resilience focusing on psychosocial processes at team level, rather than individual athletes’ traits. The reliability and validity of CREST has also been examined in Turkish athletes [36].

China, as a country, is a major sporting competitor, and has won many gold medals in the Olympic Games, but performance in team sports, such as football and basketball remains unsatisfactory. Team resilience may be one of the factors influencing team sport performance. To our knowledge, there are no studies that have focused on team resilience and its measurement in China. Ungar [37] pointed out the cultural problems in the study of psychological resilience and Gucciardi, Jackson [38] suggested that the differences between Eastern and Western cultures might reduce the universal applicability of a scale. Therefore, the purpose of this study was to further examine the reliability and validity of CREST in Chinese culture in order to assess psychological resilience in team sports.

## Methods

### Participants

A sample of 659 athletes was recruited from Chinese national, provincial, and university teams. The sample consisted of 355 male and 304 female participants, aged from 16–34 (*M* = 20.08, *SD* = 2.98). All participants have engaged in sports training for a minimum of 5 years, and had at least one competition experience in the past six months. Of all the participants, about 90% were selected from team sports, including basketball, football, volleyball, rugby, water polo, and handball; only a few participants come from tennis and archery. It should be noted that many athletes in this study come from different provinces and cities, most of informed consents were obtained by telephone. Moreover, verbal consent was also obtained from their parents for minors who participated in this study. The team coaches are witnesses when verbal consents or paper contents were authorized. Ethical approval was obtained from the Human Research Committee of Tianjin University of Sport.

### Measures

#### Team resilience

The 20-item team resilience inventory was used to measure the self-reported resilience in team sports. Consistent with recommendations for test adaptation [39], the Chinese version was developed using forward and back translation procedures by two independent translators, proficient in Chinese and English, and familiar with resilience, before measurements were taken and applied (S1 Appendix). All participants were asked to respond on a seven-point Likert scale from 1 (*strongly disagree*) to 7 (*strongly agree*). The revision has been authorised by Steven Decroos who developed CREST.

#### Effort

Intrinsic Motivation Inventory [40] was used to evaluate athlete’s perceived effort in training and competition. The subscale has three items measured by a seven-point response scale (1 = not true at all; 7 = very true). A satisfactory internal reliability and factorial validity have been demonstrated among university athletes and high school students [41].

#### Satisfaction of basic psychological needs

The Basic Psychological Needs Satisfaction in Sport Scale was used to measure participants’ three basic psychological needs. The scale has three 5-item subscales measuring autonomy, competence, and relatedness. All items are rated using a seven-point Likert scale from not true at all to very true. The scale has a good reliability and validity in a sample of Chinese athletes [42].

#### CD-RISC-10 (Chinese version)

The Connor-Davidson Resilience Scale (CD-RISC) was developed by Connor and Davidson [7]. It consists of 5 factors, 25 items with overall Cronbach’s α 0.89 and test-retest correlation 0.87 in the studies of American participants. The CD-RISC-10 was developed by Campbell-Sills and Stein [28], and Yu and Zhang [32] in China translated the CD-RISC-10 into Chinese. Each item is scored from 0 (not true at all) to 4 (true all the time). The Cronbach’s α value of the Chinese version of CD-RISC-10 is 0.91, which suggested that it is as reliable and valid as the English version to measure the individual trait resilience.

### Procedure

First, we were authorized by Steven Decroos to conduct this cross-cultural study. We then invited two independent researchers who are proficient in Chinese and English and familiar with resilience, to translate the scale using forward and back translation procedures. We obtained consent from coaches, and they were asked to send the online survey to athletes using a WeChat QR code. Coaches were asked to seek the opinions of athletes who would voluntarily participate in the survey, and give their honest responses to each item. Coaches who asked for results were provided with clear feedback. A Master’s graduate student who was familiar with resilience was responsible for the data analysis. It took approximately ten minutes for participants to complete the online survey.

### Statistical analysis

Data was analyzed in IBM SPSS Statistics 20. The factor structure was analysed with Exploratory Factor Analysis. Kaiser-Meyer-Olkin (KMO) and Bartlett’s Test of Sphericity values were calculated to determine whether the data was proper for factor analysis. Items with a factor loading of < .32, > .45, > .55, > .63, and > .71 are considered as poor, fair, good, very good, and excellent, respectively [43]. Mplus 6.0 was used to conduct confirmatory factor analysis to examine the construct validity. The normed chi-square less than 2.0 indicates a better adaptability of the model. We followed the standard for the assessment of adequate model fit, CFI and TLI > .90, RMSEA and SRMR < .08 [44]. More stringent cut-off values (CFI and TLI > .95, RMSEA and SRMR < .06) represent a good model fit [45].

## Results

Each item had a significant correlation with the total CREST score (*p* < .01). A significant difference between the first and last 27% of each item and its correlation with the total score were observed (*p* < .01), which proved good item discrimination (Table 1).

**Table 1:** The CR value and correlation between each item and total score.

Item	*CR* value	*R*	Item	*CR* value	*R*
**Q01**	5.655**	.315**	**Q11**	5.857**	.391**
**Q02**	10.543**	.559**	**Q12**	7.294**	.392 **
**Q03**	5.713**	.332**	**Q13**	11.210**	.602**
**Q04**	14.104**	.591**	**Q14**	6.670**	.414**
**Q05**	5.499**	.336**	**Q15**	12.828**	.592**
**Q06**	16.473**	.653**	**Q16**	6.669**	.372**
**Q07**	6.689**	.370**	**Q17**	7.311**	.428**
**Q08**	14.981**	.640**	**Q18**	6.086**	.367**
**Q09**	6.709**	.374**	**Q19**	14.407**	.600**
**Q10**	8.552**	.435**	**Q20**	8.840**	.466**

*CR* = critical ratio; *R* = correlation coefficient

**p < .01

Exploratory factor analysis results are presented in Table 2. KMO = .931, *p* < .001, showed that the data was suitable for exploratory factor analysis. Principal component analysis extracts two common factors with a cumulative contribution rate of variance of 61.20%. The result was consistent with the two common factors of the original scale [2]. The factor loadings ranged from .599 to .897 which showed good factorial validity and allowed for explanation based on the standard proposed by Comrey and Lee [43]. The two factors reflect the team’s ability to demonstrate resilient characteristics and vulnerabilities under pressure, and have significant correlation with CREST: DRC (r = .526, *p* < .01) and VNP (r = .687, *p* < .01). Significant correlations between positive and negative structure were also revealed (r = -.256, *p* < .01).

**Table 2:** Standardized factor loadings and communality of the Chinese version of CREST.

Items	Factor loadings	Communality	Items	Factor loadings	Communality
**Item1**	.614	.389	**Item11**	.599	.379
**Item2**	.827	.700	**Item12**	.750	.573
**Item3**	.666	.463	**Item13**	.850	.729
**Item4**	.889	.813	**Item14**	.822	.692
**Item5**	.644	.428	**Item15**	.861	.757
**Item6**	.897	.808	**Item16**	.675	.459
**Item7**	.824	.514	**Item17**	.824	.690
**Item8**	.884	.785	**Item18**	.747	.574
**Item9**	.773	.621	**Item19**	.876	.782
**Item10**	.733	.538	**Item20**	.740	.547
**Eigenvalues**	7.703	4.536			
**Cumulative variance**	38.517%	61.20%			

Extraction method: Principal Component Analysis, Rotation method: Skew Rotation Method with Kaiser Standardization

**Fig 1:**
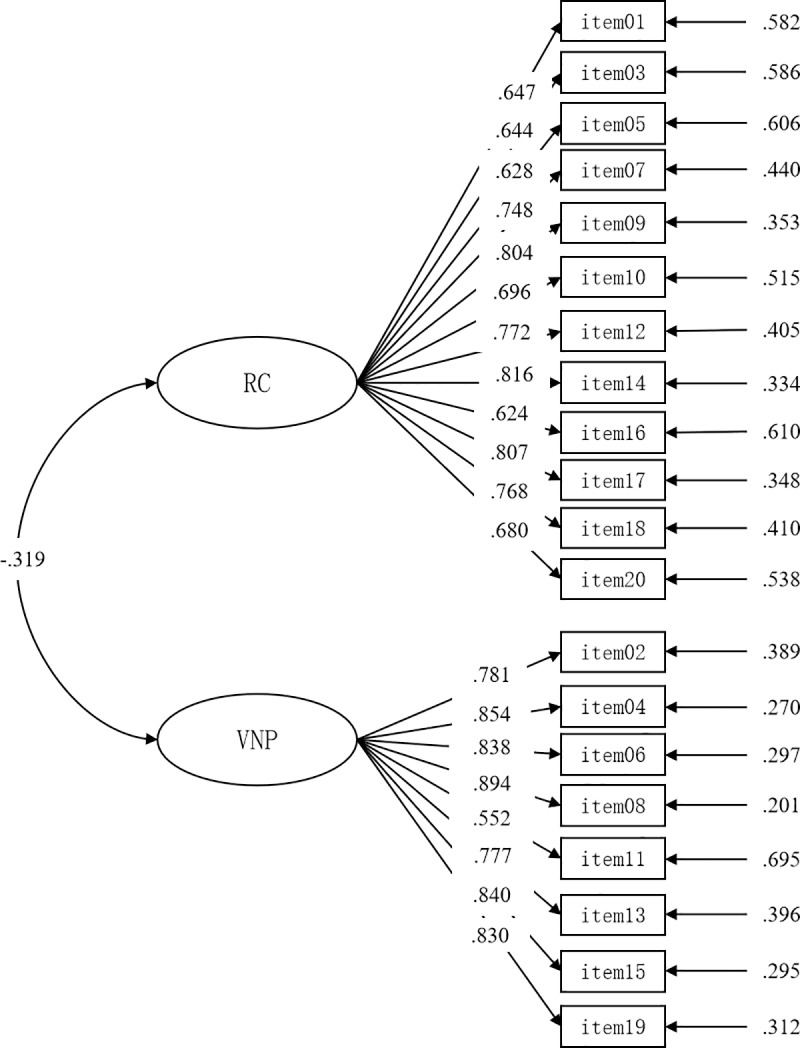
Confirmatory factor analysis results of the Chinese version of CREST. RC = resilient characteristics; VNP = vulnerabilities under pressure.

The results of Confirmatory Factor Analysis supported the two-factor measurement model: χ^2^/df = 2.481, CFI = .948, TLI = .942, RMSEA = .063, SRMR = .042. The factor loadings ranged from .552 to .894 (Fig 1). The reliability coefficient of the Chinese version of CREST was 0.842 (DRC = .927, VNP = .921). Moreover, the scale had good test-retest reliability coefficient (ICC = .836) of one-month interval. The validity was assessed by the theoretically relevant variables of the basic psychological needs satisfaction (autonomy, competence, relatedness), effort and individual resilience measured by CD-RISC-10, and was supported given the expected significant correlations between the total score of CREST, its two structures and the theoretically relevant variables (Table 3).

**Table 3:** Correlations between CREST, its two structures and theoretically relevant variables.

	Autonomy	Competence	Relatedness	Effort	CD-RISC-10
Total Score of Team Resilience	.387**	.464**	.568**	.304**	.290*
RC	.545**	.534**	.472**	.566**	.376**
VNP	-.357**	-.343**	-.368**	-.397**	-.195

RC = resilient characteristics; VNP = vulnerabilities under pressure; CD-RISC-10 = Brief Connor–Davidson Resilience Scale

*p < .05

**p < .01.

## Discussion

The purpose of this study was to examine the psychometric properties of the Characteristics of Resilience in Sports Teams inventory in Chinese team athletes. This is the first study that focused on resilience measurement in team sport in China, and the second cross-cultural study to examine the reliability and validity of CREST in different cultures. EFA results revealed a stable two-structure model of team resilience. All items had high factor loadings which ranged from 0.599 to 0.897, and showed good factorial validity in explaining team resilience.

In addition, the two-structure model of the Chinese version of CREST was further supported, RMSEA and SRMR values were lower than .80, and CFI and TLI were higher than .90. Furthermore, the factor loadings of all items of confirmatory factor analysis ranged from .552 to .894. These data supported the factorial validity of the CREST in Chinese culture, and suggested that the resilience of Chinese team athletes can be evaluated using the Chinese version of CREST. Moreover, the reliability coefficient of the Chinese version of CREST and test–retest reliability coefficient with a one-month interval also showed that the scale is reliable. These results were consistent with the original study [2] and the cross-cultural study [36].

The concurrent validity of Chinese version of CREST was also satisfying in terms of the data matching the expected correlation between team resilience and the theoretically relevant variables. The results suggested that satisfying basic psychological needs may be used as a predictor of team resilience due to the positive correlation of its three constructs with team resilience. Importantly, resilience may be improved by satisfying athletes’ basic psychological needs. Previous research revealed the positive relationship between resilience and basic psychological needs in high school students [46] and female athletes [47]. In China, athletes train in teams all year round and have little time to live with their families. The coach–athlete relationship is the most important interpersonal relationship for Chinese athletes. Research found that basic psychological needs significantly correlated with coach–athlete relationship[48] and this directly affected basic psychological needs and motivation [49]. Thus, the coach–athlete relationship may satisfy the relatedness of the basic psychological needs of athletes. Research also revealed that higher levels of relatedness significantly predicted resilience [50]. Moreover, the coach–athlete relationship is similar to that parent–child relationship in China. One athlete mentioned that “My coach is like a father to me. We started to work together when I was little and he lives in the team accommodation with me” [51]. This deep emotional relatedness can improve athletes’ resilience and help them better cope with pressure. Moreover, future researches should examined what type of coach–athlete relationship better fosters the resilient of sports teams.

On the other side, competence is the feeling that one can successfully complete highly challenging tasks [52]. High resilience athletes always believe in their ability to face and overcome pressure in a constructive way [47] and have a strong awareness of their capability. This feeling of competence will, in turn, further improve their ability to cope with pressure. Therefore, applied sport psychologists or coaches should use strategies to satisfy the basic psychological needs of athletes in order to improve their resilience.

Previous research has demonstrated the positive relationship between team resilience and effort, CD-RISC [2, 36]. Our study provides further evidence for the positive correlation between team resilience and individual resilience, and demonstrated that CD-RISC-10 can be used as a tool to assess resilience in team sport, which is consistent with previous studies [2, 35, 36]. However, there are some flaws in explaining the psychological resilience of athletes, especially team sport athletes when using CD-RISC, due to samples that came from communities, primary care outpatients, general psychiatric outpatients, clinical trial of generalised anxiety disorder, etc.

Although our study provides psychometric evidence of the Chinese version of CREST in team sports, the limitations of the study should also to be noted. First, the sample size, especially for elite athletes is not sufficient: about 30% college student athletes have few opportunities to participate in high-level competition, such as national and world-class competitions. Moreover, many athletes are not the leading players in their team, and have limited playing time in a competition season. This may affect their understanding of resilience due to lower pressure and adversity experience. Second, the samples were selected by convenience sampling, which may limit the generalization of the CREST in Chinese culture. In addition, cultural differences should be undertaken with caution in the study of resilience [37, 53]. Although CREST showed good reliability among different cultural backgrounds (China, UK, Belgium and Turkey), there were still some problems in the evaluation of Chinese team sport athletes' resilience.

Psychologists suggested that researchers should pay attention to the resilience characteristics closely related to Chinese traditional culture [54], such as Confucianism, Taoism, Buddhism and Collectivist culture. These Chinese traditional cultures may influence the response of Chinese athletes in the face of pressure and further improve the development of resilience. For example, the ideology of resilience in Confucianism emphasises hard-striving, aggressiveness, and tasting sweetness amidst bitterness during hard times. Taoism lays emphasis on compliance with nature and the world and letting things take their own course [54]. Therefore, sport psychologists should allow for Chinese traditional cultures in developing measurement tools of resilience in team sport.

In conclusion, this study demonstrates the reliability and validity of the Chinese version of CREST when assessing the characteristics of resilience in Chinese team sports. However, due to limitations mentioned above, future study should be cautious when using the scale to assess athletes’ resilience. We will further examine the psychometric properties of the translated Chinese version of CREST among world-class athletes, and develop a team resilience measurement tool based on Chinese traditional culture in the future.

## Supporting information

S1 Appendix The Chinese version of CREST. (DOCX)

S1 Data (RAR)
