# Supplementary material for: Psychometric evaluation of the characteristics of resilience in sports team inventory in China
Source: PLoS One. 2020 Jun 12;15(6):e0234134. doi: 10.1371/journal.pone.0234134 (PMC7292377; doi:10.1371/journal.pone.0234134)
Supplement: S1 Appendix — (DOCX) [file pone.0234134.s001.docx]

Dear, players！

Thank you for participating in this research on the resilience of sports teams.

The following statements describe some of the possible characteristics that sports teams may display when they experience pressure. Please keep in mind those moments that your team wasunder pressure in the past monthand indicate the extent to which you agree or disagree with the following statements. “1” represents ‘strongly disagree’, “7" represents ‘strongly agree’.

Your answers will allow us to better understand the way in which sports teams deal with pressuring situations. Therefore, your honesty is very important for this research. Moreover, your answers will remain strictly confidential and will never be passed on to your coach or others. Participating in this study is voluntary. You can abort your participation at any time.

**English Editon**

| **In the past month, when my team was under pressure…** | **strongly disagree** | |  | **neutral** |  | **strongly**  **agree** | |  |
| --- | --- | --- | --- | --- | --- | --- | --- | --- |
| the team was able to focus on what was important | 1 | 2 | 3 | 4 | 5 | 6 | 7 |  |
| teammates started to communicate negatively with each other | 1 | 2 | 3 | 4 | 5 | 6 | 7 |  |
| team members fought for each other | 1 | 2 | 3 | 4 | 5 | 6 | 7 |  |
| the team lost its confidence | 1 | 2 | 3 | 4 | 5 | 6 | 7 |  |
| I felt that I could count on other members of the team | 1 | 2 | 3 | 4 | 5 | 6 | 7 |  |
| the level of collective effort in the team dropped | 1 | 2 | 3 | 4 | 5 | 6 | 7 |  |
| effective communication kept players’ minds focused on  the!task!at!hand | 1 | 2 | 3 | 4 | 5 | 6 | 7 |  |
| team members started to mistrust one another | 1 | 2 | 3 | 4 | 5 | 6 | 7 |  |
| members of the team were committed to contributing to the collective  belief!of!the!team | 1 | 2 | 3 | 4 | 5 | 6 | 7 |  |
| team members fought hard to not let each other down | 1 | 2 | 3 | 4 | 5 | 6 | 7 |  |
| individuals forgot their role in the team and did not know what they had to do   1. to!do! | 1 | 2 | 3 | 4 | 5 | 6 | 7 |  |
| the challenges we have gone through as a team helped us learn to withstand pressures   1. withstand!pressures | 1 | 2 | 3 | 4 | 5 | 6 | 7 |  |
| there came no support from teammates | 1 | 2 | 3 | 4 | 5 | 6 | 7 |  |
| the strong bonds between teammates helped the team during difficult times   1. during!difficult!times | 1 | 2 | 3 | 4 | 5 | 6 | 7 |  |
| the team could not persist through the most difficult moments | 1 | 2 | 3 | 4 | 5 | 6 | 7 |  |
| the team was able to reset their focus to alleviate pressure | 1 | 2 | 3 | 4 | 5 | 6 | 7 |  |
| the team gained belief by working together to withstand pressures   1. withstand!pressures | 1 | 2 | 3 | 4 | 5 | 6 | 7 |  |
| the team drew on an agreed team vision, values, and guiding behavioural principles   1. behavioural!principles | 1 | 2 | 3 | 4 | 5 | 6 | 7 |  |
| the team did not belief in its ability to withstand pressure | 1 | 2 | 3 | 4 | 5 | 6 | 7 |  |
| the team reflected on a shared team vision | 1 | 2 | 3 | 4 | 5 | 6 | 7 |  |

**Chinese Editon**

亲爱的运动员，您好！

欢迎参加我们关于运动团队心理弹性的调查研究。

下面一些问题代表了一个运动团队在面临压力时可能表现出的特点。请注意：过去几个月你所在的队伍经历了压力，然后请您仔细回想过去几个月之内，你的运动队在面临压力时的表现，并在相应的数字上划“√”，其中1代表“非常不同意”，7代表“非常同意”。

| **在过去几个月的比赛中, 当队伍面临压力的时候** | **非常**  **不同意** | |  | **中 等** |  | **非常**  **同意** | |  |
| --- | --- | --- | --- | --- | --- | --- | --- | --- |
| 队伍能够集中注意力在重要的事情上面 | 1 | 2 | 3 | 4 | 5 | 6 | 7 |  |
| 队友相互之间的交流开始变得消极 | 1 | 2 | 3 | 4 | 5 | 6 | 7 |  |
| 队友们为彼此而战 | 1 | 2 | 3 | 4 | 5 | 6 | 7 |  |
| 这个队伍失去了他的信心 | 1 | 2 | 3 | 4 | 5 | 6 | 7 |  |
| 我感觉我可以从团队其他队员那里获得帮助 | 1 | 2 | 3 | 4 | 5 | 6 | 7 |  |
| 队伍不再像以前那么齐心协力 | 1 | 2 | 3 | 4 | 5 | 6 | 7 |  |
| 有效的沟通让队员保持注意集中在当前任务上 | 1 | 2 | 3 | 4 | 5 | 6 | 7 |  |
| 队伍成员彼此之间开始不信任 | 1 | 2 | 3 | 4 | 5 | 6 | 7 |  |
| 队伍成员以能够为团队的集体信念做出贡献为己任 | 1 | 2 | 3 | 4 | 5 | 6 | 7 |  |
| 队伍成员们为了不让彼此失望都在努力奋斗 | 1 | 2 | 3 | 4 | 5 | 6 | 7 |  |
| 个别运动员忘记了他们在团队中的角色，不知道他们应该做什么 | 1 | 2 | 3 | 4 | 5 | 6 | 7 |  |
| 团队经历的挑战帮助队员们学会如何承受压力 | 1 | 2 | 3 | 4 | 5 | 6 | 7 |  |
| 我感觉缺少队友们的支持 | 1 | 2 | 3 | 4 | 5 | 6 | 7 |  |
| 队友之间牢固的纽带帮助队伍渡过困难时期 | 1 | 2 | 3 | 4 | 5 | 6 | 7 |  |
| 队伍可能在最困难的时刻无法坚持下去 | 1 | 2 | 3 | 4 | 5 | 6 | 7 |  |
| 队伍能够重新调整注意力来缓解压力 | 1 | 2 | 3 | 4 | 5 | 6 | 7 |  |
| 通过团队协作，队员们应对压力的信念能够提高 | 1 | 2 | 3 | 4 | 5 | 6 | 7 |  |
| 队伍的团队目标、价值观和行为指导原则达成了一致 | 1 | 2 | 3 | 4 | 5 | 6 | 7 |  |
| 队伍没有信心应对压力 | 1 | 2 | 3 | 4 | 5 | 6 | 7 |  |
| 队伍有共同的团队目标 | 1 | 2 | 3 | 4 | 5 | 6 | 7 |  |
